# Computational Systems Biology of Alfalfa – Bacterial Blight Host-Pathogen Interactions: Uncovering the Complex Molecular Networks for Developing Durable Disease Resistant Crop

**DOI:** 10.3389/fpls.2021.807354

**Published:** 2022-02-17

**Authors:** Raghav Kataria, Naveen Duhan, Rakesh Kaundal

**Affiliations:** ^1^Department of Plants, Soils, and Climate, College of Agriculture and Applied Sciences, Utah State University, Logan, UT, United States; ^2^Bioinformatics Facility, Center for Integrated Biosystems, Utah State University, Logan, UT, United States; ^3^Department of Computer Science, College of Science, Utah State University, Logan, UT, United States

**Keywords:** host-pathogen interactions, domain-domain, alfalfa, *Pseudomonas syringae* ALF3, type III secretion system, effectors, bacterial stem blight, ice-nucleation proteins (INP)

## Abstract

*Medicago sativa* (also known as alfalfa), a forage legume, is widely cultivated due to its high yield and high-value hay crop production. Infectious diseases are a major threat to the crops, owing to huge economic losses to the agriculture industry, worldwide. The protein-protein interactions (PPIs) between the pathogens and their hosts play a critical role in understanding the molecular basis of pathogenesis. *Pseudomonas syringae* pv. *syringae* ALF3 suppresses the plant’s innate immune response by secreting type III effector proteins into the host cell, causing bacterial stem blight in alfalfa. The alfalfa-*P. syringae* system has little information available for PPIs. Thus, to understand the infection mechanism, we elucidated the genome-scale host-pathogen interactions (HPIs) between alfalfa and *P. syringae* using two computational approaches: interolog-based and domain-based method. A total of ∼14 M putative PPIs were predicted between 50,629 alfalfa proteins and 2,932 *P. syringae* proteins by combining these approaches. Additionally, ∼0.7 M consensus PPIs were also predicted. The functional analysis revealed that *P. syringae* proteins are highly involved in nucleotide binding activity (GO:0000166), intracellular organelle (GO:0043229), and translation (GO:0006412) while alfalfa proteins are involved in cellular response to chemical stimulus (GO:0070887), oxidoreductase activity (GO:0016614), and Golgi apparatus (GO:0005794). According to subcellular localization predictions, most of the pathogen proteins targeted host proteins within the cytoplasm and nucleus. In addition, we discovered a slew of new virulence effectors in the predicted HPIs. The current research describes an integrated approach for deciphering genome-scale host-pathogen PPIs between alfalfa and *P. syringae*, allowing the researchers to better understand the pathogen’s infection mechanism and develop pathogen-resistant lines.

## Introduction

Alfalfa (*Medicago sativa* L.) or lucerne, a member of the Fabaceae family, is a common forage legume in the United States and other countries. Because of its broad adaptation, high biomass production, and perennial nature, it is known as the “Queen of forages.” It also aids in soil and water conservation, biological nitrogen fixation, and insect pest interruption in crop rotations, making it an important component of sustainable agriculture (Nemchinov et al., 2017). The crop is widely cultivated in the regions of hot, dry summers and well-drained soils, and thus is successfully grown in similar environments of Asia Minor, Iran, Southern Europe, Mexico, and the Middle East (Putnam and Orloff, 2014). As per the reports of the USDA National Agricultural Statistics Service (NASS), the estimated value of alfalfa hay and haylage produced in the United States counts to $10.8 billion per year, with an area of 11.7 million acres under production and an average yield of 3.61 tons per acre in 2019^1^.

Many diseases affect alfalfa, but a recent outbreak of bacterial stem blight in the western and central United States has caused concern. There have also been cases of the disease recorded in Europe, Australia, and western Iran (Harighi, 2007). The disease, caused by *Pseudomonas viridiflava*, was first reported in California and Utah in April and May of 2016 and 2017, with the symptoms highly similar to those caused by *Pseudomonas syringae* pv. *syringae*, which causes a significant loss to crop quantity and quality (Lipps et al., 2019). *P. syringae* pv. *syringae*, a ubiquitous epiphyte, is a major disease-causing pathogen in a wide variety of cultivated plant species. It is a rod-shaped gram-negative bacterium with a well-sequenced genome. *P. syringae* species have a broad variety of virulence factors, such as a type III secretion system (T3SS), ice nucleation activity, toxic substances, cell wall degrading enzymes, and exopolysaccharides, which makes it a good model for better understanding the pathogen-host interactions (Morris et al., 2013). The strains of *P. syringae* produce a phytotoxin, syringomycin, which is thought to play a role in the virulence of the pathogen. Most strains are known to function as ice nuclei, causing frost damage to plants when temperatures drop below zero (Agrios, 2005). The disease infection occurs in two phases- localized foliar necrosis (blight) and systemic vascular wilt. The bacterium infects the host stem mainly at frost injury sites, where it penetrates and causes water-soaked lesions, as well as the emergence of spindly stems that blacken with age. Depending on the incidence rate, the disease can result in yield losses of up to 50% or more (Race et al., 2006; Lamichhane et al., 2015). The outer membrane (Lindow et al., 1989) of *P. syringae* is surrounded by a unique protein, ice-nucleation protein (INP), that mimics the crystalline structure of ice and thus serves as an initiator for ice formation, linking bacterial stem blight to frost. This protein is of high interest to the researchers not only because of its pathogenicity, but also because of its other potential applications such as frozen food preparation and snowmaking. Scientists also discovered that the disease-causing strain of *P. syringae* is a weak pathogen of crops such as sugar beet and snap bean, and a few genes that are unique to the alfalfa pathogen have been identified (Li Q. et al., 2012; Proud sponsor of Midwest Forage Association, 2017).

Most of the disease-causing mechanisms include protein-protein interactions (PPIs), which play an important role in the infection process as well as in initiating the defense responses against the disease. Therefore, studying the PPI network between the pathogen and plant proteins is a crucial step for understanding the underlying mechanism of the infection (Loaiza et al., 2020). Genomic sequencing reveals that nearly 3,000 proteins directly interact with the pathogen proteins or are involved in plant defense (Bishop et al., 2000). On a genome-wide scale, the computational approaches reveal the relationships between predicted proteins. Recently, a wide range of computational methods for predicting host-pathogen interactions (HPIs) have been developed, based on diverse data types or properties such as protein sequence similarity (Matthews et al., 2001; Sun et al., 2017), protein domain interactions (Ng et al., 2003; Binny Priya et al., 2013), protein structural information (Keskin et al., 2008), and gene ontology (GO) annotations (Wu et al., 2006; Zhong and Rajapakse, 2020). Among the available computational methods, the interolog and domain-based methods (Shoemaker and Panchenko, 2007) are most used.

In the present study, we deciphered genome-wide PPIs between *Medicago sativa* and *Pseudomonas syringae* pv. *syringae* ALF3 using interolog (homology-based) and domain-based methods. We discovered that functional characterization of the HPIs can reveal the molecular mechanisms of pathogen infection. Furthermore, we identified novel protein hubs, enriched molecular function, biological processes, and pathways by conducting extensive functional annotation of the predicted interactome, which could be crucial in fully understanding disease infection mechanism. We also predicted the localization of *P. syringae* pv. *syringae* ALF3 protein in alfalfa cells. We assume that the predicted virulence factors described in this study will serve as a solid foundation for future experimental validations and provide a deeper understanding of pathogen infection in alfalfa.

## Materials and Methods

**FIGURE 1:**
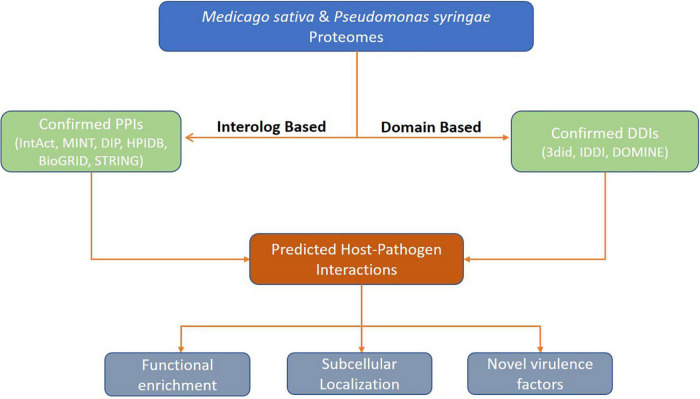
Overall framework for prediction of protein-protein interactions between Alfalfa and *Pseudomonas syringae*.

A detailed pipeline of the computational prediction and functional analysis of HPIs is depicted in Figure 1.

### Data Collection

The whole proteomes of *M. sativa* and *P. syringae* pv. *syringae* strain ALF3 were downloaded from different sources. *M. sativa* proteome was downloaded from LegumeIP v3 (Dai et al., 2020), consisting of 87,892 protein sequences, while proteome of *P. syringae* was downloaded from JGI IMG/M (Taxon ID: 2609460275) and UniProt database (UP000028706)^2^ with a count of 4,983 and 4,842 protein sequences, respectively. The redundant protein sequences were removed from the proteomes using CD-HIT (Fu et al., 2012) at 100% identity. In the case of *P. syringae*, the proteomes from both the sources were merged, followed by the removal of duplicate sequences.

Proteins whose subcellular position was predicted to be a cytoplasmic membrane or cytoplasm membrane were excluded from our analysis because they are not thought to be involved in host-pathogen interactions. Other proteins classified as periplasmic, extracellular, outer membrane, or unknown were considered to be positive candidates for interactions. Following that, the entire *P. syringae* proteome was analyzed in the EffectiveDB^3^ (Jehl et al., 2011) to predict the proteins that are labeled as secreted for the proteins predicted as cytoplasmic. These proteins were also thought to be promising candidates for pathogen-host interactions. After removing the redundancy and cytoplasmic proteins in the proteomes, HPIs were identified using 87,156 alfalfa and 4,427 *P. syringae* protein sequences.

To infer the interolog predictions, the analysis was implemented in inter- as well intra-species interaction databases, namely IntAct v4.2.16 (Kerrien et al., 2012), MINT 2018 (Molecular INTeraction Database) (Licata et al., 2012), HPIDB v3.0 (Host-Pathogen Interaction Database) (Kumar and Nanduri, 2010), DIP 2020 (Database of Interacting Proteins) (Salwinski et al., 2004), BioGRID v4.2.191 (Biological General Repository for Interaction Datasets) (Chatr-Aryamontri et al., 2017), and interactions for 66 plant species from STRING v11.0 (Search Tool for the Retrieval of Interacting Genes/Proteins) (Szklarczyk et al., 2019). Sequences from all the databases were downloaded and local blast databases were created for each.

To implement the domain-based prediction, three domain-domain interaction (DDI) databases, namely 3did v2020_01 (three-dimensional interacting domains) (Mosca et al., 2014), DOMINE v2.0 (Database of Protein Domain Interactions) (Raghavachari et al., 2008), and IDDI v2011.05.16 (Integrated Domain-Domain Interaction) (Kim et al., 2012) were downloaded and stored in individual files. For analysis, the Pfam v31.0 (Finn et al., 2014) database was downloaded to predict the domains for host and pathogen proteins. Further, using the Pfam database, the identified domains for the host and pathogen species were queried against the three databases (3did, DOMINE and IDDI) to search for the domain-domain interactions. Detailed information about the databases, along with the number of sequences and interactions is available in Supplementary Material 1, excel sheet 1.

### Computational Prediction of Protein-Protein Interactions Between Alfalfa and *Pseudomonas*

#### Interolog-Based Prediction

Interolog is the method of determining the conserved interactions between two proteins based on their sequence similarity (Yu et al., 2004). When two interacting proteins X and Y in one species have interacting orthologs X’ and Y’ in another, the interaction pairs X-Y and X’-Y’ are referred to as interologs. The proteomes of *M. sativa* and *P. syringae* were aligned against six protein-protein interaction databases, viz. IntAct, MINT, DIP, HPIDB, BioGRID, STRING, using BLASTp v2.7.1 with default parameters (*e*-value, sequence identity, sequence coverage) to make the prediction. An optimal combination of parameters to filter the BLAST alignments of *M. sativa* and *P. syringae* was determined using *e*-value (1e-04, 1e-05, 1e-10, 1e-20, 1e-25, 1e-30, 1e-50), sequence identity (30, 40, 50, and 60%), and sequence coverage (30, 40, 50, 60, and 80%). However, no gold standard cutoff criteria for the prediction of HPIs is reported. In the past, researchers predicted the cassava protein interactome using the interolog-based approach with at least 60% identity, 80% sequence overlap, and an *e*-value lower than 1e-10 (Thanasomboon et al., 2017). In another study on Human-*Burkholderia pseudomallei* (Loaiza et al., 2020), they used an *e*-value of 1e-50, 40% sequence identity, and 80% sequence coverage to decode the host-pathogen relationship. The investigation on Arabidopsis-*Pseudomonas syringae* (Sahu et al., 2014) for the prediction of plant-pathogen interactions used the *e*-value of 1e-04, sequence identity of 50%, and sequence coverage of 80%. In our study, we discovered that a sequence identity of at least 30%, an *e*-value of less than 1e-10, and sequence coverage of at least 80% was the best combination of alignment parameters for predicting HPIs.

#### Domain-Based Prediction

The domain-based approach takes into account the protein domain profiles of known intra-species PPIs, allowing it to predict host-pathogen protein-protein interactions (Dyer et al., 2007). The prediction in domain-based methods is based on protein structural knowledge found in various domain-domain interaction (DDI) databases. The host and pathogen domains were inferred from the Pfam database using HMMER v3.3.1 (Eddy, 2011), which were then used to predict PPIs using in-house SQL queries on the local machine. For *M. sativa*, the results of hmmscan were filtered using an *e*-value and coverage of 1e-23 and 0.2, respectively, while for *P. syringae*, an *e*-value and coverage of 1e-18 and 0.35 were used. Hmmscan has been successfully used in a number of studies to infer host-pathogen interactions (Mondal et al., 2017; Cuesta-Astroz et al., 2019; Lian et al., 2020).

#### Integration of Interolog- and Domain-Based Approaches

After separately implementing interolog and domain methods as described above, the results from both the approaches were merged, and duplicate predictions were eliminated. Since there were a high number of predicted PPIs (more than 10 million), we considered the consensus predictions of both approaches, which reduces the chances of false-positive HPIs. A similar approach of consensus interactions has been considered previously while predicting HPIs from multiple interaction databases (Loaiza et al., 2020).

### Subcellular Localization of Proteins Involved in Host-Pathogen Interactions

Plant pathogenic bacteria relies on type III secretion systems to infect host cells, thus affecting a variety of host cell mechanisms (Aung et al., 2017; Xue et al., 2019). So, to understand the infection mechanism of the pathogen, subcellular localization analysis is an essential step in the study. We evaluated various bioinformatics methods to determine the subcellular position of the proteins, and an appropriate benchmark was chosen for the study, based on the maximum positive matches with the subcellular localization annotation at UniProt^4^. We used PSORTb 3.0.2 (Yu et al., 2010), a commonly used tool for bacterial protein localization, to determine the subcellular localization of *P. syringae* proteins. To determine the subcellular localization of alfalfa proteins, a homology-based and machine learning model of the tool Plant-mSubP (Sahu et al., 2020) was implemented with default parameters.

### Dataset Collection for Known Effectors in *P. syringae* for Validation

The effectors are deployed by the pathogenic bacteria to inhibit the plant immune response and disrupt the host cell mechanisms (Pelgrom et al., 2020). In this regard, we extracted effector proteins, T3SS-hop proteins, and virulence proteins from EffectiveDB (see text footnote 3), *Pseudomonas syringae* Genome Resources^5^, and Pseudomonas genome DB^6^, respectively. The following criteria was followed to obtain effector proteins from the above-mentioned sources.

#### Potential Effector Proteins

EffectiveDB was used to predict the possible effector proteins of *P. syringae*, using *P. syringae* pv. *syringae* B728 as a reference. EffectiveDB is a database of bacteria predicted secreted proteins that includes five methods for effector prediction (EffectiveT3, EffectiveELD, EffectiveCCBD, Predotar, and T4SEpre). EffectiveELD, EffectiveCCBD, and EffectiveT3 proteins were chosen because these methods predict “type III secreted” proteins. A total of 641 effector proteins were predicted.

#### Type III Secretion System-Hop Proteins

The known T3SS effector proteins belonging to *P. syringae* were obtained from *Pseudomonas syringae* Genome Resources. In literature (Dillon et al., 2019), it has been reported that there are 66 effector proteins, but we found only 26 effectors which were further processed.

#### Virulence Proteins

The virulence factor proteins were downloaded from Pseudomonas genome DB using *Pseudomonas aeruginosa PAO1* as reference. The orthologs were searched and eight virulence factors were obtained for *P. syringae*.

In total, we obtained 655 unique effectors (known, potential, and virulence factors) combined from the above-mentioned sources. These effectors were also used in the selection of an appropriate combination of parameters for filtering BLAST alignments.

### Dataset Collection for Ice-Nucleation Proteins for Validation

Ice-nucleation proteins are associated with bacterial stem blight as these are thought to be the initiator of the frost mechanism. The gene, *Psyr1608*, located on the outer membrane of the bacteria, belongs to *Pseudomonas syringae* pv. *syringae* B728a (Feil et al., 2005). This gene is known to contain the octamer repeat of the bacterial ice-nucleation proteins, and is thus involved in the disease. Taking this into consideration, we found the INPs in the projected HPIs. We obtained 30 orthologs of the INPs from Pseudomonas genome DB (see text footnote 6), which were then BLASTed against the *P. syringae* ALF3 protein dataset. We filtered the BLAST alignments at 40% identity to achieve high performance, which reduced the number of INPs to 17. These were then identified in the predicted HPIs.

### Functional Enrichment Analysis

In order to classify the proteins in HPIs that have a common function/biological pathway, it is necessary to obtain the functional annotation of the proteins involved in the interactions. The R package clusterProfiler (Yu et al., 2012) was used to conduct Gene Ontology (GO) and Kyoto Encyclopedia of Genes and Genomes (KEGG) analyses. Benjamini and Hochberg test correction method (Benjamini and Hochberg, 1995) was used to calculate the adjusted *p*-values, and enriched GO terms were filtered based on adjusted *p*-value cutoff of ≤0.05. All the GO annotation ontologies (cellular component, molecular function, and biological processes) were used for gene ontology enrichment analysis. A GO database for alfalfa proteins was created using the *makeOrgPackage* function in the R package “AnnotationForge,” and GO enrichment analysis of alfalfa proteins was performed, while *P. syringae* proteins GO terms were enriched with *org.Psyringae.eg.db* Bioconductor package. KEGG pathway enrichment for alfalfa and *P. syringae* proteins was performed using enrichKEGG function in ClusterProfiler package at a *p*-value cutoff of 0.05.

### Visualization of Interaction Networks and Identification of Protein Hubs

The study of protein-protein interaction network help in a deeper understanding of various cellular mechanisms involved in the process. To visualize the PPI networks, Gephi v0.9.2 (Bastian et al., 2009) was used in combination with various layout algorithms such as Hu (2006), OpenOrd (Martin et al., 2011), and others to improve the readability of the network (Pavlopoulos et al., 2017). We also analyzed the network on different parameters such as betweenness, closeness, and degree. Functional enrichment analysis of combined interactions is present in Supplementary Material 6 and Supplementary Figures 1–5. Effector analysis of combined interactions is present in Supplementary Material 7.

## Results and Discussion

**FIGURE 2:**
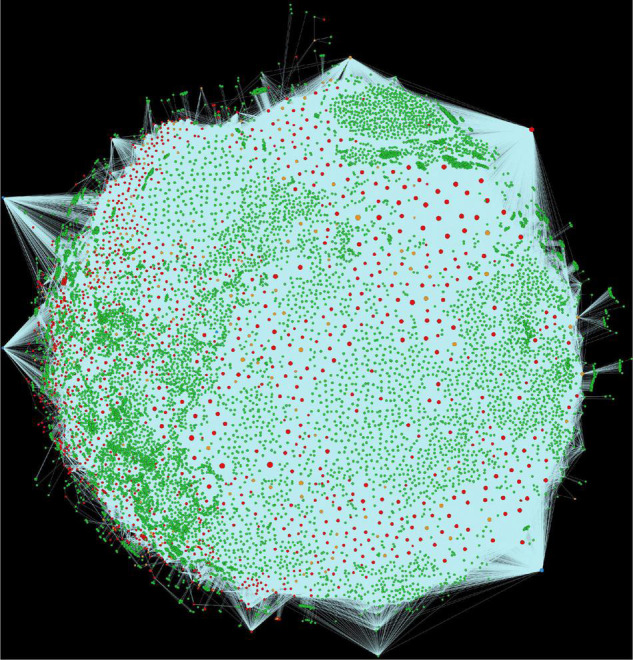
Visualization of the complete consensus interactions for alfalfa-*P. syringae* interaction system. Green nodes are host proteins, red nodes are pathogen proteins, orange nodes are effectors, and blue nodes are ice nucleation proteins. Edges in light green represent the consensus interactions from both interolog and domain-based methods.

Alfalfa and *P. syringae* proteomes were randomly paired to identify the genome-wide protein-protein interactions. The interaction probability of each pair was estimated individually through computational approaches: domain-based and interolog-based models (Table 1). The interactome, combined from both the computational approaches, contained a total of 14,186,848 putative PPIs, involving 50,629 alfalfa and 2,932 *P. syringae* proteins (details of these HPIs is presented in Supplementary Material 2, excel sheets 3–17). To reduce the number of false-positive interactions we selected 690,634 non-redundant HPIs predicted by both the approaches, involving 10,935 alfalfa and 1,386 *P. syringae* proteins, named as common interactions throughout the manuscript (detail of these HPIs is presented in Supplementary Material 3, excel sheet 3). The visualization of the common network is shown in Figure 2. We present a thorough review of protein hubs and the functional enrichment of proteins found in the “common” subnetwork in the following sections.

**TABLE 1:** Host-pathogen protein-protein interactions predicted using orthology and domain-based methods for the Alfalfa-*P. syringae* interaction system.

Method	Number of interactions	Host proteins	Pathogen proteins
**Interolog-based**			
IntAct	216,185	9,645	1,560
MINT	20,934	2,842	519
HPIDB	3,037	1,153	306
BioGRID	415,450	11,543	1,911
DIP	8,147	1,795	581
STRING	11,760,939	49,973	1,348
**Total (Interolog)**	**11,916,848**	**49,995**	**2,202**
**Domain-based**			
3did	86,622	8,476	1,695
IDDI	2,574,097	13,693	2,430
DOMINE	1,052,624	9,540	1,780
**Total (Domain)**	**2,960,634**	**14,328**	**2,515**
Interolog + Domain (combined)	14,186,848	50,629	2,932
Interolog + Domain (consensus)	690,634	10,935	1,386
Interolog (unique)	11,226,214	49,993	2,192
Domain (unique)	2,270,000	14,134	2,497

*Total (Interolog): The predicted HPIs from all the six interolog databases were merged and duplicates were removed.*

*Total (Domain): The predicted HPIs from all the three domain databases were merged and duplicates were removed.*

*Interolog + Domain (combined): The predicted HPIs from both the methods were merged and the duplicates were removed.*

*Interolog + Domain (consensus): For both the methods, given the large number of interactions, the consensus of the predicted HPIs was preferred, to minimize the presence of false positives.*

*Interolog (unique): The unique HPIs containing the interactions only from interolog-based method.*

*Domain (unique): The unique HPIs containing the interactions only from domain-based method.*

### Protein Hubs

Protein network analysis is widely implied to investigate the significant nodes in an extensive network (Ashtiani et al., 2018). Simultaneous interactions between PPI hubs and proteins have been observed in biological networks. Identifying the role of such interactions could further bolster the understanding of the infection mechanism of the pathogen (Yang et al., 2019). In this study, we discovered a large number of protein hubs. Protein hubs are an important component of a HPI network, providing deep insights into various biological pathways, molecular processes, cellular biochemistry, and physiology (Kuzmanov and Emili, 2013), which is useful in deciphering the virulence mechanism of a host-pathogen system. The detailed analysis information on protein hubs is provided in Supplementary Material 4, excel sheets 1, 2. Despite the fact that hubs were determined based on node degree, a greater collection of topological measures were calculated to provide a more detailed portrait of the information. The following is a brief description of the values of certain topological measures within the common network.

#### Degree

**FIGURE 3:**
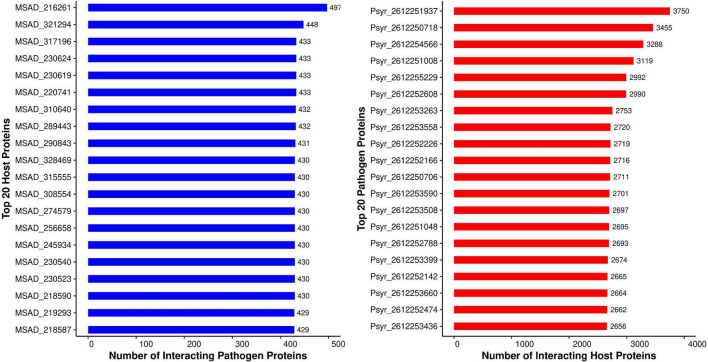
Top 20 host (blue) and pathogen (red) hubs identified in the *Medicago sativa-P. syringae* protein-protein interaction system.

The metric used in this study to evaluate hubs and nodes was the degree. The *P. syringae* proteins had an average degree of 498, while the alfalfa proteins had an average degree of 63. Pathogen proteins, as predicted, have a higher degree (and a larger betweenness) than other proteins in the popular subnetwork. The ratio of the interacting proteins in common network is coherent with previous reports of computational PPI prediction, whereby a small number of pathogen proteins interact with the host interactome (Li Z. G. et al., 2012; Kurubanjerdjit et al., 2013). Studies in the past have established that during the process of infection in the host, a pathogen extensively mutates itself. While on the other side, the plant expands its gene families in response to the pathogen attack (Stahl and Bishop, 2000). Thus, the reason for the ratio of interacting proteins being maintained throughout. We reported a large number of protein hubs in this study; the top 20 hubs from *P. syringae* as well as the alfalfa are discussed below. Summary of the 20 hub nodes for *P. syringae* and alfalfa proteins, as well as the number of their interacting partners are presented in Figure 3.

#### Closeness Centrality

The closeness centrality of a node in a network shows how close it is to all other nodes in the network. The average of the shortest path lengths from the node to every other node in the network is used to measure it. The average closeness centrality value for alfalfa and *P. syringae* proteins in the common network was 0.333 and 0.332, respectively. The highest closeness centrality value of 1 was found between five alfalfa proteins (MSAD_214560, MSAD_257396, MSAD_258406, MSAD_298516, MSAD_310524) and 11 *P. syringae* proteins (Psyr_2612254914, Psyr_2612251287, Psyr_2612254775, Psyr_2612254957, Psyr_2612251834, Psyr_2612252057, Psyr_ 2612254066, Psyr_2612251255, Psyr_2612253067, Psyr_26 12254813, Psyr_2612255124).

#### Betweenness Centrality

The betweenness centrality measures how close a node is to other nodes. The number of shortest paths that pass through the target node is used to calculate this metric. The average betweenness centrality value for alfalfa and *P. syringae* proteins in the common network was 8.34e-3 and 1.49e-2, respectively. The highest betweenness centrality value of 1 was found between 1 alfalfa protein (MSAD_321294) and 1 *P. syringae* protein (Psyr_2612250718).

#### *Medicago sativa* Hubs

The protein hubs network revealed that the strongly interacting host protein (MSAD_216261) was discovered to be cyclic nucleotide-binding domain (CNBD) protein. CNBD proteins are mostly involved in pathogen effector (avirulence proteins) recognition, which further leads to downstream signaling activation, and hence pathogen resistance (DeYoung and Innes, 2006). A study on Arabidopsis experimentally identified 12 cyclic nucleotide binding proteins, of which eight proteins contain the cyclic nucleotide binding domain, and these proteins are involved in hydrogen peroxide signaling and immune response (Donaldson et al., 2016). The protein, MSAD_321294, was found to be sensitive to aluminum rhizotoxicity. Aluminum toxicity has been shown to reduce root growth, and alteration in abscisic acid levels in root apices indicate its role in the plant response to aluminum toxicity (Kopittke, 2016). MSAD_220741, a chaperone protein encoded by the DnaK gene, was present in the top 20 host hubs. This gene is involved in plant response to various stresses. In rice seedlings, DnaK was shown to be upregulated in response to heavy metal stress (Ul Haq et al., 2019). DnaK regulates hydrogen peroxide production and ABA-induced antioxidant response during heat and drought stress (Yu et al., 2015). The majority of the top 20 alfalfa hub proteins were found to be heat shock proteins, classified into heat shock cognate 70 kDa proteins (MSAD_230624, MSAD_317196, MSAD_230523, MSAD_230540, MSAD_245934, MSAD_ 256658, MSAD_219293), and heat shock 70 kDa protein (MSAD_289443, MSAD_310640, MSAD_290843, MSAD_218590, MSAD_274579, MSAD_308554, MSAD_ 315555, MSAD_328469, MSAD_218587). Heat shock cognate 70 (Hsc) proteins are involved in protein folding and intracellular targeting, while the heat shock 70 (Hsp70) proteins are involved in signal transduction and protein translocation during abiotic and biotic stresses (Kominek et al., 2013). Hsp70s play a role in both infection mechanisms and the host response to stress. Studies show that Hsp70 is the primary target for *P. syringae* virulence effector, HopI1. HopI1 hijacks plant Hsp70 and localizes itself to the chloroplast (Jelenska et al., 2010).

#### *P. syringae* Hubs

The superfamily II DNA and RNA helicases, which include *Pseudomonas* proteins Psyr_2612251937 and Psyr_2612254566, form the largest pathogen hub in the common interaction network. Superfamily II helicases are divided into DEAD and DExH box families based on the sequence of a conserved motif (Steimer and Klostermeier, 2012). These helicases play an essential role in bacterial replication. Studies in *E. coli* reveal that absence of DEAD-box helicase leads to growth defects under laboratory conditions (Jagessar and Jain, 2010). The pathogen protein “Psyr_2612250718” is a member of the Major Facilitator Superfamily (MFS) transporter family, which is ubiquitously found in prokaryotes. A MFS transporter (*mfsG*) was discovered in *Botrytis cinerea* that was involved in isothiocyanate (ITC) detoxification and showed upregulation on interaction with wild-type Arabidopsis *in planta* (Vela-Corcía et al., 2019). Five proteins (Psyr_2612255229, Psyr_2612253263, Psyr_2612252226, Psyr_2612252166, Psyr_2612252474) in the common network were identified as 3-oxoacyl-(acyl-carrier-protein) reductase (OAR). OAR is responsible for catalyzing 3-oxoacyl-ACP reduction in the fatty acid synthesis pathway. In *Pseudomonas aeruginosa* PAO1, 12 OAR-encoding genes have been identified, which play a key role in the production of specific quorum-sensing signals in the bacteria (Guo et al., 2019). The pathogen proteins (Psyr_2612253558, Psyr_2612250706, Psyr_2612253590, Psyr_2612252788, Psyr_2612252142) be-longed to the NAD(P)-dependent dehydrogenase family. A novel NADP+-dependent D-arabitol dehydrogenase enzyme, identified from rust fungus *Uromyces fabae*, showed increased concentration during pathogenesis and quenched reactive oxygen species in the host immune response (Link et al., 2005). Another chaperone protein Psyr_2612252608, encoded by HtpG, was found in the common network. High-temperature protein G (HtpG), a Hsp90 homolog, is reported to be involved in the cell protection against environmental stress (Grudniak et al., 2015).

### Functional Enrichment Analysis

Functional enrichment analysis is a critical step in determining the biological importance of proteins involved in host and pathogen PPIs. We examined the functional compositions of the respective proteins by analyzing GO and KEGG pathways enrichment. The existence of enriched (over-represented) functional categories were found to be closely linked to host defense and pathogen infection validates predicted HPIs.

#### Gene Ontology

To determine the significant functions of the alfalfa and *P. syringae* proteins involved in PPIs, the proteins were investigated using GO enrichment analysis based on three functional categories of gene ontology viz. cellular part, molecular function, and biological process. The enrichment was performed using enrichment score [−log10(*P*-value)] of the GO terms.

**FIGURE 4:**
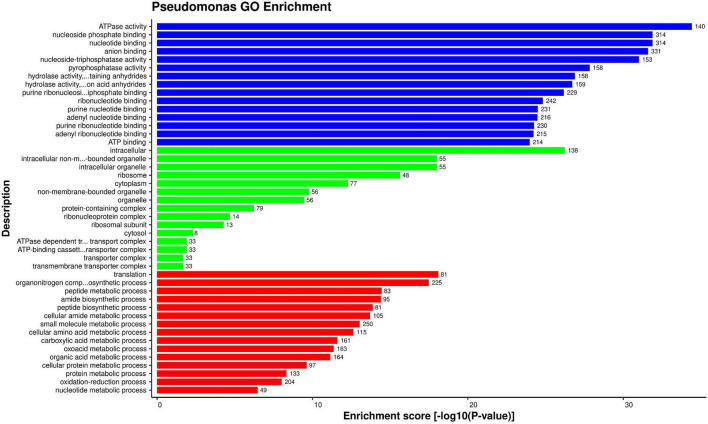
Top 15 *P. syringae* GO terms that were found over-represented based on enrichment score [–log 10(*P*-value)]: Molecular function (blue), cellular component (green), and biological process (red).

A total of 573 GO terms were found to be enriched for *P. syringae* proteins in the interactions. The detailed enrichment analysis data is present in Supplementary Material 4, excel sheet 4. The most enriched GO terms in the cellular component category were GO:0043229 (intracellular organelle), GO:0043232 (intracellular non-membrane-bounded organelle), and GO:0005840 (ribosome). In the molecular function category, GO:0000166 (nucleotide binding), GO:1901265 (nucleoside phosphate binding), and GO:0016887 (ATPase activity) were found to be most enriched. While in the biological process category, GO:0006412 (translation), GO:0055114 (oxidation-reduction process), and GO:0019538 (protein metabolic process) were found to be abundant. Top 15 most enriched GO terms from all three categories are depicted in Figure 4. Aside from these, a number of GO terms (GO:0071944, GO:0030313, GO:0031975, GO:0009279) were linked to the pathogen’s external membrane and the ice-nucleation gene (Psyr1608). This gene has also been found in *Pseudomonas syringae* pv. *syringae* B728a, which aids in ice crystal formation (Feil et al., 2005). *P. syringae* protein enrichment revealed the importance of the host for pathogens to carry out different molecular and biological processes.

**FIGURE 5:**
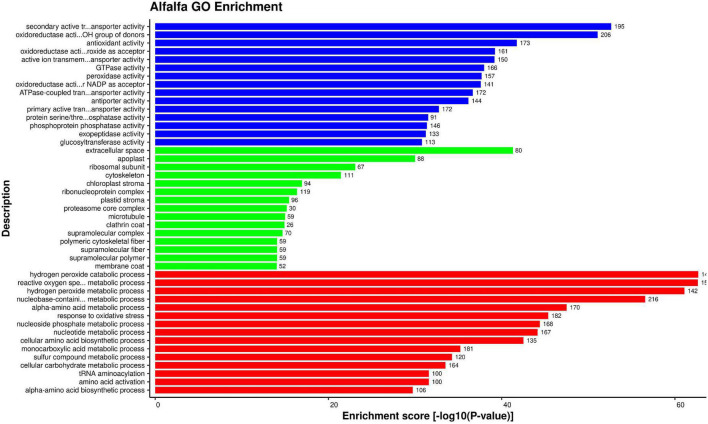
Top 15 Alfalfa GO terms that were found over-represented based on enrichment score [–log 10(*P*-value)]: Molecular function (blue), cellular component (green), and biological process (red).

For alfalfa proteins, 2127 GO terms were enriched. The detailed enrichment analysis data is present in Supplementary Material 4, excel sheet 3. The most enriched GO terms in biological process were GO:0070887 (cellular response to chemical stimulus), GO:0006979 (response to oxidative stress), and GO:0032870 (cellular response to hormone stimulus). The highly enriched GO terms for cellular component were GO:0005794 (Golgi apparatus), GO:0005618 (cell wall), and GO:0030312 (external encapsulating structure). In molecular function category, the most enriched terms were GO:0016614 (Oxidoreductase activity), GO:0015291 (secondary active transmembrane transporter activity), and GO:0015297 (antiporter activity). For each category top 15 enriched GO terms are depicted in Figure 5.

**FIGURE 6:**
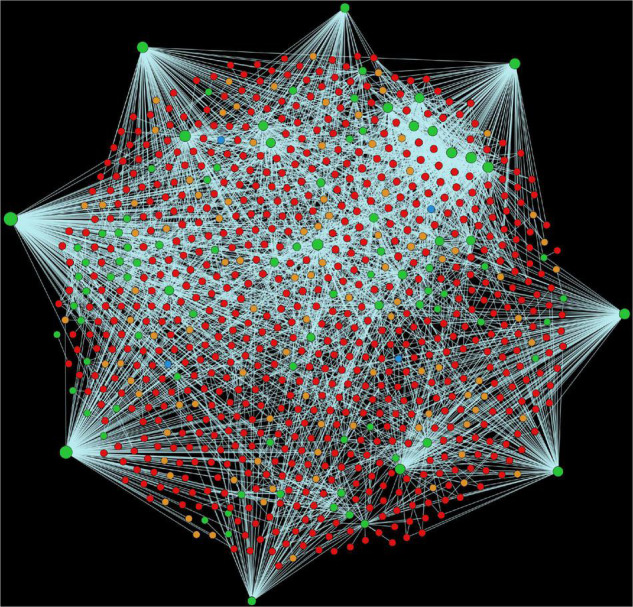
Visualization of the GO term GO:0009570 (Chloroplast stroma). Green nodes are host proteins, red nodes are pathogen proteins, orange nodes are effectors, and blue nodes are ice nucleation proteins. Edges in light green represent the consensus interactions from both interolog and domain-based methods.

Chloroplast stroma (GO:0009570) was also found to be enriched in alfalfa proteins. A total of 3,382 interactions were discovered whereby 94 alfalfa proteins were found to be interacting with 754 pathogen proteins (Figure 6). It has previously been established that homologs of many components of bacterial signal recognition particle (SRP) pathways are present in the chloroplast. These SRP systems assist in the transport of proteins to the thylakoid membrane, which serves as a bacterial infection site (Ziehe et al., 2017). Chloroplast also serves as a production house of various defense-related signals [reactive oxygen species (ROS)] and hormones [abscisic acid (ABA), jasmonic acid (JA)], which play a crucial role in the immune response of plants. These are a primary site for the pathogen effectors to attack and overcome plant immune signaling (Park et al., 2018). ABA synthesis occurs in the chloroplast and is an essential plant growth regulator that induces stomatal closure in response to pathogen attack, thereby restricting pathogen entry (Lu and Yao, 2018). ROS contributes to the induction of hypersensitive response (HR) and cell wall strengthening (Kretschmer et al., 2020). Reduced ROS production in the chloroplast caused the reduction in HR in tobacco on the attack with *Xanthomonas campestris* pv. *vesicatoria* (Zurbriggen et al., 2009).

Protein phosphorylation (GO:0006468) was found to be involved in about 39 alfalfa proteins. These proteins interact with 395 pathogen proteins, owing to 2,701 PPIs. Phosphorylation is considered as one of the most common post-translational modifications, and about one-third of all eukaryotic proteins are believed to be phosphorylated (Olsen et al., 2006), which usually occurs on threonine (Thr) and serine (Ser) residues (de la Fuente van Bentem and Hirt, 2009). In past, phosphorylation has been demonstrated to play a role in the generation of immune responses in plants (Park et al., 2012). In Arabidopsis, the treatment of cell cultures with flg22 (flagellin peptide) or xylanase (fungal elicitor) lead to the identification of 1,170 novel phosphorylation sites from 472 phosphoproteins, which evoke an early immune response (Benschop et al., 2007), thus indicating the differential phosphorylation of proteins and the importance of phosphorylation in plant immunity. Additionally, the interaction networks generated for top biological process (GO:0042744; hydrogen peroxide catabolic process), and molecular function (GO:0015291; secondary active transmembrane transporter activity) are depicted in Supplementary Figures 6, 7, respectively.

#### Kyoto Encyclopedia of Genes and Genomes Pathway Enrichment

**FIGURE 7:**
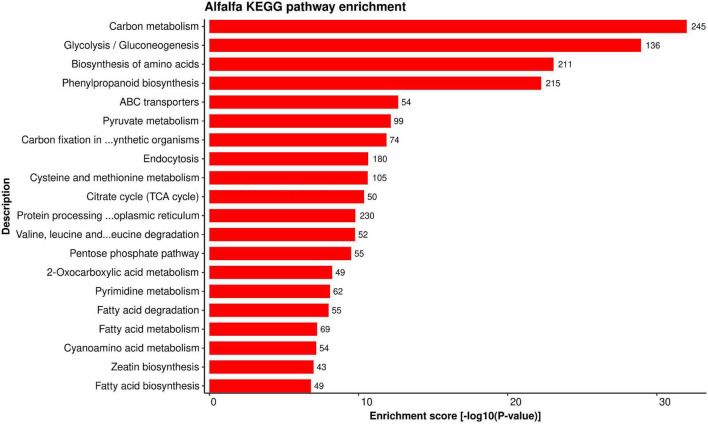
Top 20 Alfalfa KEGG pathways that were found over-represented in the HPIs based on enrichment score [–log 10(*P*-value)].

**FIGURE 8:**
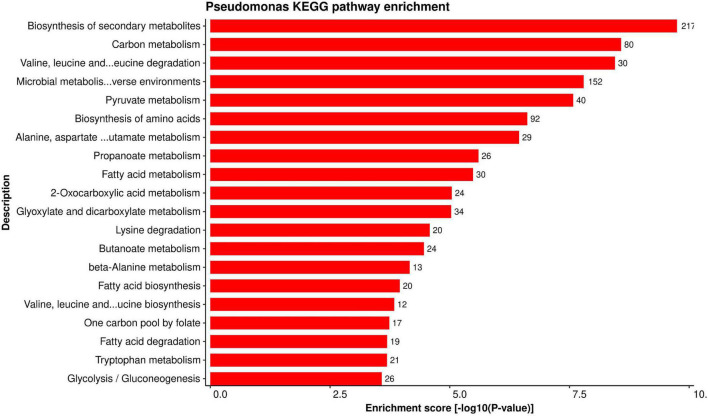
Top 20 *P. syringae* KEGG pathways that were found over-represented in the HPIs with the lower *P*-values (0.05) are shown.

Establishing a connection between the role of important genes (and/or proteins) and specific pathway provides more information about the importance of a gene in various mechanisms. KEGG pathway enrichment analysis was performed to better understand the biological aspects of the proteins in the predicted HPI network. We found 116 KEGG pathways enriched for alfalfa proteins versus 86 pathways enriched for *P. syringae* proteins in total. The detailed pathway enrichment analysis data is present in Supplementary Material 4, excel sheets 5, 6 for alfalfa and *P. syringae*, respectively. Top 20 enriched KEGG pathways for alfalfa and *P. syringae* proteins are depicted in Figures 7, 8, respectively.

**FIGURE 9:**
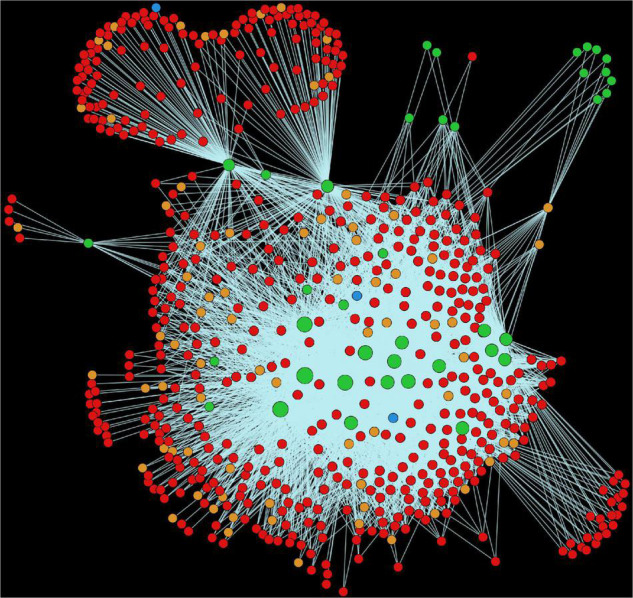
Visualization of the KEGG pathway mtr04626 (Plant pathogen interaction). Green nodes are host proteins, red nodes are pathogen proteins, orange nodes are effectors, and blue nodes are ice nucleation proteins. Edges in light green represent the consensus interactions from both interolog and domain-based methods.

Plant-pathogen interaction (mtr04626) is a substantially enriched pathway in the host-pathogen interaction system. In this pathway, 55 alfalfa proteins and 1,838 *Pseudomonas* proteins are involved in 27,253 interactions. Unlike animals, plants have multiple defense layers against the invading pathogens. These include different levels of responses such as PAMP-triggered immunity (PTI) being the primary response, while effector-triggered immunity (ETI) is known as the secondary. In *Petunia*, the plant-pathogen interaction pathway was found to be enriched in both pollinated and unpollinated corollas, and the defense-related genes (mostly interacting with Ca^2+^) in this pathway showed upregulation during flower senescence (Broderick et al., 2014). From the network (Figure 9), it was analyzed that the host protein, MSAD_331284 (with the highest degree), interacts with 396 proteins. Besides plant-pathogen interaction (mtr04075), this protein is also involved in a variety of plant defense-related pathways, including the MAPK signaling pathway (mtr04016) and plant hormone signal transduction. The protein was also found to interact with one of the ice-nucleation proteins (INPs), which serve as the nuclei for ice formation, and hence initiates the disease.

ABC transporters pathway (mtr02010) was found to be associated with 54 alfalfa proteins. ABC transporters play a significant role in plant-pathogen interactions (Kang et al., 2011). Pleiotropic drug resistance (PDR), a subfamily of ABC transporters, is associated with plant defense mechanisms. A study on Arabidopsis discovered a gene (*AtPDR12*) that putatively encodes PDR, and the expression of this gene against the pathogen requires sensitivity to jasmonates and salicylic accumulation, both of which play an essential role in plant immunity (Campbell et al., 2003). In another study, sclareolide (an antifungal diterpene) was used to treat the cell cultures of *Nicotiana plumbaginifolia*, which resulted in identification of *NpABC1* gene that encodes ABC transporter and is also related to the secretion of secondary metabolites associated with plant defense. *NpABC1*-encoded protein was discovered to be localized in the plasma membrane (Jasiński et al., 2001).

**FIGURE 10:**
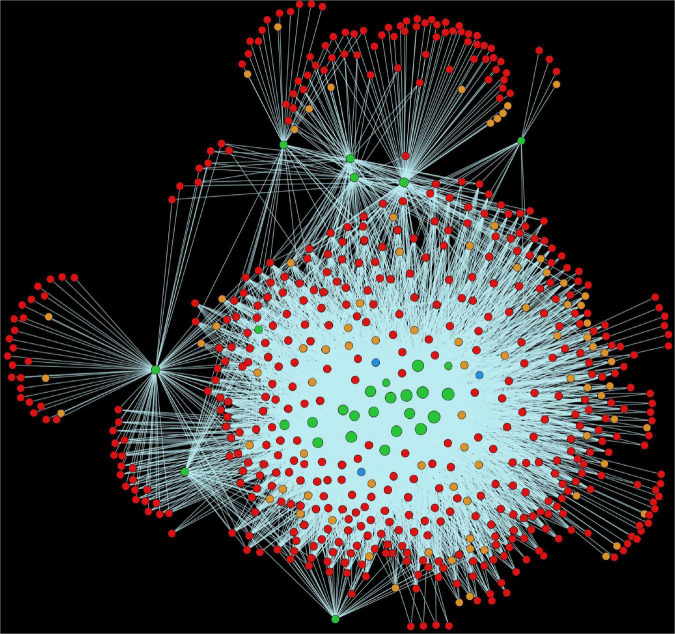
Visualization of the KEGG pathway mtr04016 (MAPK signaling pathway). Green nodes are host proteins, red nodes are pathogen proteins, orange nodes are effectors, and blue nodes are ice nucleation proteins. Edges in light green represent the consensus interactions from both interolog and domain-based methods.

In the MAPK signaling pathway (mtr04016), we found 102 alfalfa proteins involved in 5,566 interactions with 597 pathogen proteins (Figure 10). Mitogen-activated protein kinases (MAPKs) are implicated in signal transduction in response to plant stress and development (Bigeard and Hirt, 2018). It was reported that *BWMK1*, a rice MAPK, showed rapid activation of protein kinase activity in presence of different pathogen signals such as salicylic acid, jasmonic acid, ethylene, and hydrogen peroxide (Cheong et al., 2003). Plant defense signaling was further demonstrated by the activation of two MAPKs, SIPK and WIPK, in *Nicotiana tabacum* in response to pathogen-related signals and various abiotic stresses, thus indicating the role of MAPK in plant stress response (Seo et al., 1995). The activation of the orthologs of SIPK and WIPK in alfalfa and Arabidopsis has been observed under stress conditions (Zhang and Klessig, 1997; Nühse et al., 2000).

A total of 305 proteins in alfalfa are linked to protein processing in endoplasmic reticulum (mtr04141). The endoplasmic reticulum (ER) is considered as the production site for secretory proteins (Yamada et al., 2011), and regulates hormone biosynthesis (Eichmann and Schäfer, 2012). The interference of plant physiological conditions with protein folding or accumulation of misfolded proteins in the ER results in ER stress. ER quality control system regulates such conditions and generates the unfolded protein response (UPR), leading to the elimination of misfolded proteins from the secretory pathway or upregulation of components required for protein folding. UPR in plants is mediated by specific membrane-associated transcription factors such as the bZIP family (bZIP17 and bZIP28) (Srivastava et al., 2012). In Arabidopsis, a few transmembrane bZIP transcription factors (AtbZIP17, AtbZIP28, and AtbZIP60), residing in the ER have been reported (Urade, 2009; Liu and Howell, 2010), which are engaged in response to ER stress. Beta-Alanine metabolism (mtr00410) involves 49 alfalfa proteins, which has a key role in various plant metabolisms, and also acts as a defense compound against various physiological stresses in plants (Parthasarathy et al., 2019). Transcriptomic studies in Arabidopsis have identified a considerable number of genes involved in amino acid metabolism against avirulent *Pseudomonas syringae* pv. tomato (*AvrRpt2*) (Scheideler et al., 2002). Despite of reduced glutamine, alanine, and proline levels than wild type plants, Arabidopsis mutant lysine histidine transporter 1 (*lht1*) showed enhanced resistance to various fungal, oomycete, and bacterial pathogens (Rojas et al., 2014). The majority of alfalfa proteins are involved in plant hormone signal transduction (mtr04075), thus controlling the plant growth and development in response to biotic and abiotic stresses (Schepetilnikov and Ryabova, 2017). Several metabolic pathways were also found to be linked to the predicted proteins in HPIs, including pyruvate metabolism (mtr00620), glycolysis/gluconeogenesis (mtr00010), glycerophospholipid metabolism (mtr00564), and fatty acid metabolism (mtr00564) (mtr01212).

On the other hand, *P. syringae* proteins are involved in secondary metabolite biosynthesis (psb01110). Secondary metabolites in pathogens have been shown to mimic plant effector molecules (such as auxins and abscisic acid) and subvert the plant defense mechanism (Pusztahelyi et al., 2015). Furthermore, pathogen proteins are found to be enriched in ABC transporters pathway (psb02010). This class of protein families is suggested to be important for phytopathogenesis (Zeng and Charkowski, 2020).

The predicted HPIs using computational methods contain possible candidates for experimental validation of the HPIs, which further provide insights into the infection mechanisms of *P. syringae*, according to the functional enrichment.

### Subcellular Localization

**FIGURE 11:**
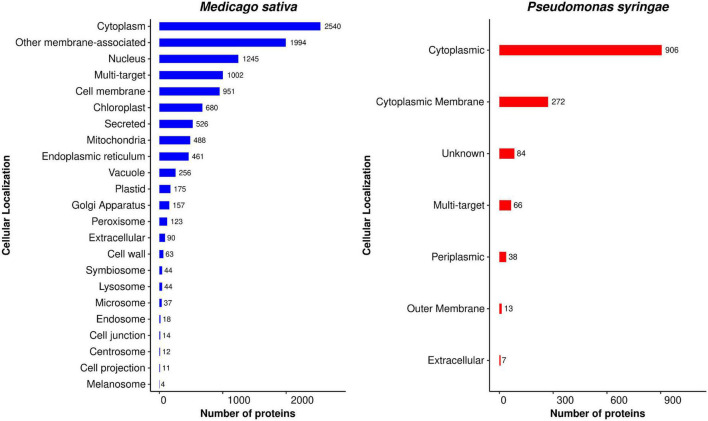
Distribution of subcellular localization of host proteins (blue) and pathogen proteins (red) involved in the predicted interactions (consensus from interolog and domain-based methods).

A pathogen directs effector proteins to the cytoplasm of host cells in order to inhibit host immunity. These effector proteins are carried to various subcellular locations after crossing the host plasma membrane and destabilize the host immune system, allowing pathogen development (Zalguizuri et al., 2018). Predicting the subcellular localization of *P. syringae*-targeted alfalfa proteins may thus provide insight into HPI mechanisms. The presence of targeted proteins in cellular components, a possible site for pathogen infection in an organism, supports this theory. We predicted the subcellular localization of alfalfa and *P. syringae* proteins to gain a better understanding of the position of host-pathogen interactions in the cell. The distribution of the subcellular localization of the predicted proteins in alfalfa and *P. syringae* is depicted in Figure 11, and the detailed list is available in Supplementary Material 5, excel sheets 1, 2. The subcellular localization of the proteins involved in the common interactions revealed that 23.22% of alfalfa proteins are localized in the cytoplasm, 18.23% are membrane-associated. While in *Pseudomonas*, 65.37% of proteins are localized in the cytoplasm and 19.62% in the plasma membrane. The results indicate that most of the interactions between host and pathogen proteins occur in the cytoplasmic region and membranes.

We were also curious to see where the *P. syringae* proteins were found after the pathogen had multiplied in the alfalfa cells. As a result, we determined the distribution of alfalfa protein localization categories for each interacting pathogen localization class. For example, 906 cytoplasmic proteins from *P. syringae* interacted with 10,004 alfalfa proteins in 491,034 interactions, out of which 24.23% (2,424 of 10,004 in 71,469 interactions) are localized in cytoplasm followed by other membrane-associated 18.03% (1,804 of 10,004 in 130,352), nucleus 11.55% (1,156 of 10,004 in 38,728 interactions) multi-target 9.61% (962 of 10,004, in 43,944 interactions), and 8.05% (806 of 10,004 in 74,576 interactions) as cell membrane (Supplementary File 5 excel sheets 1, 2). In the multi-target (66 out 1,386 in 42,395 interactions) category, the majority of alfalfa proteins were found to be targeted toward other membrane, cytoplasm, cell membrane and nucleus category. This shows that the cytoplasmic pathogen proteins can interact inside the cytoplasm or in other membrane or nucleus of the host cell. Similarly, cytoplasmic membrane proteins (272) from *P. syringae* interacted with alfalfa 7,385 proteins in 134,597 interactions, which are localized in 23.39% (1,728/7,385 in 53,528 interactions) other membrane-associated, followed by cytoplasm 18.06% (1,334/7,385 in 11,900 interactions), cell membrane 11.11% (821/7,385 in 29,529 interactions), nucleus 9.24% (683/7,385 in 12,138 interactions), and 8.66% (640/7,385 in 9,321 interactions) as multi-target, etc. The 38 *P. syringae* periplasmic proteins interacted with 2,807 alfalfa proteins in 6,766 interactions, out of which 25.50% (716/2,807 in 1,482 interactions) were localized in other membrane-associated followed by cytoplasm 14.03% (394/2,807 in 1,590 interaction), and nucleus 11.72% (329/2,807 in 672 interactions), etc. The seven *P. syringae* proteins interacted with 1,965 alfalfa protein in 2,550 interactions, out of which 23.05% (453/1,965 in 592 interactions) were localized in other membrane-associated followed by cytoplasm 15.01% (295/1,965 in 346 interaction), and chloroplast 11.85% (233/1,965 in 406 interactions), etc. From these results, we can infer that *P. syringae* proteins mostly interacted with other membrane-associated alfalfa proteins followed by cytoplasm, cell membrane, nucleus, multi-target chloroplast.

### Effectors and Ice Nucleation Proteins

In the common predictions, 192 effectors and four INPs were identified in the interactions. These proteins were found to be involved in various pathways, including biosynthesis of secondary metabolites (psb01110), carbon metabolism (psb01200), pyruvate metabolism (psb00620), glycolysis/gluconeogenesis (psb00010), citric (TCA) cycle (psb00020), and microbial metabolism in diverse environments (psb01120). The identified proteins were also associated with various GO annotations, viz., protein metabolic process (GO:0019538), gene expression (GO:0010467), cellular protein modification process (GO:0006464), cytoplasm (GO:0005737), transferase activity (GO:0016740), etc.

### Identification of Novel Host Targets for Known and Potential Effectors of *P. syringae*

The novel host targets for known effectors were identified by searching the known 26 effectors in the consensus predictions, and it was found that four effectors interact with 1,787 host proteins, involved in 1,787 interactions. More than half of the identified host proteins were found to be the members of ABC transporter and kinase families, including LRR-receptor-like kinase, calmodulin-binding receptor-like cytoplasmic kinase, MAP kinase, calcium-dependent kinase, calcium-dependent protein kinase (CDPK), serine/threonine kinase, and others. DEAD-box RNA helicase was annotated in 56 host proteins. In rice, a gene (*OsBIRH1*) encoding a DEAD-box RNA helicase protein was cloned, which showed activated expression on treatment with defense-related signals in seedling leaves. Also, the *OsBIRH1*-overexpressing transgenic Arabidopsis plants were tolerant to oxidative stress (Li et al., 2008).

To identify the novel host targets for potential effectors, 646 potential effectors were searched in the consensus predictions, which identified 190 effectors with 8,266 host interacting partners, involved in 94,243 interactions. The host proteins were classified as disease resistance protein, DEAD-box RNA helicase, LRR and NB-ARC domain disease resistance, kinases, glycoside hydrolase family 1, and ubiquitin-conjugating enzyme. In higher plants, glycoside hydrolase 1 (GH1) is known to play an important role in plant defense against various biotic and abiotic stresses, as well as other processes such as lignification, cell wall hydrolysis (Xu et al., 2004). Ubiquitination has a great importance in response to plant defense against pathogens. A study in wheat identified a ubiquitin-conjugating enzyme (TaU4), which is localized in nucleus and cytoplasm, and acts as a negative regulator against *Zymoseptoria tritici* (Millyard et al., 2016).

### Identification of Novel Virulence Effectors

Additionally, we searched for the novel *P. syringae* effectors in the predicted HPIs. For this, we analyzed the *P. syringae* protein dataset on EffectiveDB at default parameters. From EffectiveDB, we identified around 3,927 *P. syringae* proteins which were classified as EffectiveT3, EffectiveCCBD, and EffectiveELD. These proteins were then identified in our predicted HPIs and it was found that these effectors count for 686,184 interactions. We obtained 1,371 novel virulence effectors interacting with 10,934 host proteins. Each pathogen protein was found to interact with multiple host proteins.

### Interolog and Domain Combined Interactions

The interactome, combined from both the computational approaches, contained a total of 14,186,848 putative PPIs, involving 50,629 alfalfa and 2,932 *P. syringae* proteins. The domain-based approach predicted 2,960,634 interactions, involving of 14,328 alfalfa and 2,515 *P. syringae* proteins, while the interolog-based approach predicted 11,916,848 interactions, involving of 49,995 alfalfa and 2,202 *P. syringae* proteins. Supplementary Material 2, excel sheets 1, 2, contains a catalog of both host and pathogen proteins, as well as their sequences. Due to the large number of interactions and computational limitations, we were unable to generate/visualize the protein-protein interaction network. Functional enrichment analysis revealed that enriched GO terms are consistent with enriched terms from the “common” interactions as discussed above.

## Data Availability Statement

The datasets presented in this study can be found in online repositories. The names of the repository/repositories and accession number(s) can be found in the article/Supplementary Material.

## Author Contributions

RKau formulated and designed the research and contributed to supervision, project administration, and funding acquisition. RKat analyzed the data, developed prediction models, and performed functional enrichment analysis, validations, literature mining, and contributed to writing—original draft preparation. RKau and ND contributed to writing—review and editing. RKat, ND, and RKau contributed to visualization. All authors have read and agreed to the published version of the manuscript.

## Conflict of Interest

The authors declare that the research was conducted in the absence of any commercial or financial relationships that could be construed as a potential conflict of interest.

## Publisher’s Note

All claims expressed in this article are solely those of the authors and do not necessarily represent those of their affiliated organizations, or those of the publisher, the editors and the reviewers. Any product that may be evaluated in this article, or claim that may be made by its manufacturer, is not guaranteed or endorsed by the publisher.

## Footnotes

1
https://www.nass.usda.gov/

2
https://www.uniprot.org/proteomes/

3
https://effectors.csb.univie.ac.at/

4
https://www.uniprot.org/

5
http://pseudomonas-syringae.org/

6
https://pseudomonas.com/

## Supplementary Material

Supplementary Figure 1 Top 15 *P. syringae* GO terms that were found over-represented based on enrichment score [−log 10(*P*-value)]: Molecular function (blue), cellular component (green), and biological process (red).

Supplementary Figure 2 Top 15 Alfalfa GO terms that were found over-represented based on enrichment score [−log 10(*P*-value)]: Molecular function (blue), cellular component (green), and biological process (red).

Supplementary Figure 3 Top 20 Alfalfa KEGG pathways that were found over-represented in the HPIs based on enrichment score [−log 10(*P*-value)].

Supplementary Figure 4 Top 20 *P. syringae* KEGG pathways that were found over-represented in the HPIs with the lower *P*-values (0.05) are shown.

Supplementary Figure 5 Distribution of subcellular localization of host proteins (blue) and pathogen proteins (red) involved in the predicted interactions (consensus from interolog and domain-based methods).

Supplementary Figure 6 Visualization of the GO term GO:0042744 (hydrogen peroxide catabolic process). Green nodes are host proteins, red nodes are pathogen proteins, and orange nodes are effectors. Edges in light green represent the consensus interactions from both interolog and domain-based methods.

Supplementary Figure 7 Visualization of the GO term GO:0015291 (secondary active transmembrane transporter activity). Green nodes are host proteins, red nodes are pathogen proteins, orange nodes are effectors, and blue nodes are ice nucleation proteins. Edges in light green represent the consensus interactions from both interolog and domain-based methods.

## References

[B1] Agrios G. N. (2005). “Chapter twelve–plant diseases caused by prokaryotes: bacteria and mollicutes,” in *Plant Pathology (5th Edn)*, ed. Agrios G. N. (San Diego, CA: Academic Press), 615–703. 10.1016/b978-0-08-047378-9.50018-x

[B2] Ashtiani M. Salehzadeh-Yazdi A. Razaghi-Moghadam Z. Hennig H. Wolkenhauer O. Mirzaie M. (2018). A systematic survey of centrality measures for protein-protein interaction networks. *BMC Syst. Biol.* 12:80. 10.1186/s12918-018-0598-2 30064421 PMC6069823

[B3] Aung K. Xin X. Mecey C. He S. Y. (2017). Subcellular localization of *pseudomonas* syringae pv. Tomato effector proteins in plants. *Methods Mol. Biol.* 1531 141–153. 10.1007/978-1-4939-6649-3_12 27837488 PMC5643156

[B4] Bastian M. Heymann S. Jacomy M. (2009). “Gephi: an open source software for exploring and manipulating networks,” in *Proceedings of the International AAAI Conference on Weblogs and Social Media*, San Jose, CA, 361–362.

[B5] Benjamini Y. Hochberg Y. (1995). Controlling the false discovery rate: a practical and powerful approach to multiple testing. *J. R. Stat. Soc. Ser. B* 57 289–300. 10.1111/j.2517-6161.1995.tb02031.x

[B6] Benschop J. J. Mohammed S. O’Flaherty M. Heck A. J. R. Slijper M. Menke F. L. H. (2007). Quantitative phosphoproteomics of early elicitor signaling in *Arabidopsis*. *Mol. Cell. Proteomics* 6 1198–1214. 10.1074/mcp.M600429-MCP200 17317660

[B7] Bigeard J. Hirt H. (2018). Nuclear signaling of plant MAPKs. *Front. Plant Sci.* 9:469. 10.3389/fpls.2018.00469 29696029 PMC5905223

[B8] Binny Priya S. Saha S. Anishetty R. Anishetty S. (2013). A matrix based algorithm for protein-protein interaction prediction using domain-domain associations. *J. Theor. Biol.* 326 36–42. 10.1016/j.jtbi.2013.02.016 23473859

[B9] Bishop J. G. Dean A. M. Mitchell-Olds T. (2000). Rapid evolution in plant chitinases: molecular targets of selection in plant-pathogen coevolution. *Proc. Natl. Acad. Sci. U.S.A.* 97 5322–5327. 10.1073/pnas.97.10.5322 10805791 PMC25827

[B10] Broderick S. R. Wijeratne S. Wijeratn A. J. Chapin L. J. Meulia T. Jones M. L. (2014). RNA-sequencing reveals early, dynamic transcriptome changes in the corollas of pollinated petunias. *BMC Plant Biol.* 14:307. 10.1186/s12870-014-0307-2 25403317 PMC4245787

[B11] Campbell E. J. Schenk P. M. Kazan K. Penninckx I. A. M. A. Anderson J. P. Maclean D. J. (2003). Pathogen-responsive expression of a putative atp-binding cassette transporter gene conferring resistance to the diterpenoid sclareol is regulated by multiple defense signaling pathways in *Arabidopsis*. *Plant Physiol.* 133 1272–1284. 10.1104/pp.103.024182 14526118 PMC281622

[B12] Chatr-Aryamontri A. Oughtred R. Boucher L. Rust J. Chang C. Kolas N. K. (2017). The BioGRID interaction database: 2017 update. *Nucleic Acids Res.* 45 D369–D379. 10.1093/nar/gkw1102 27980099 PMC5210573

[B13] Cheong Y. H. Moon B. C. Kim J. K. Kim C. Y. Kim M. C. Kim I. H. (2003). BWMK1, a rice mitogen-activated protein kinase, locates in the nucleus and mediates pathogenesis-related gene expression by activation of a transcription factor. *Plant Physiol.* 132 1961–1972. 10.1104/pp.103.023176 12913152 PMC181281

[B14] Cuesta-Astroz Y. Santos A. Oliveira G. Jensen L. J. (2019). Analysis of predicted host–parasite interactomes reveals commonalities and specificities related to parasitic lifestyle and tissues tropism. *Front. Immunol.* 10:212. 10.3389/fimmu.2019.00212 30815000 PMC6381214

[B15] Dai X. Zhuang Z. Boschiero C. Dong Y. Zhao P. X. (2020). LegumeIP V3: from models to crops—an integrative gene discovery platform for translational genomics in legumes. *Nucleic Acids Res.* 49 D1472–D1479. 10.1093/nar/gkaa976 33166388 PMC7778993

[B16] de la Fuente van Bentem S. Hirt H. (2009). Protein tyrosine phosphorylation in plants: more abundant than expected? *Trends Plant Sci.* 14 71–76. 10.1016/j.tplants.2008.11.003 19162527

[B17] DeYoung B. J. Innes R. W. (2006). Plant NBS-LRR proteins in pathogen sensing and host defense. *Nat. Immunol.* 7 1243–1249. 10.1038/ni1410 17110940 PMC1973153

[B18] Dillon M. M. Almeida R. N. D. Laflamme B. Martel A. Weir B. S. Desveaux D. (2019). Molecular evolution of *Pseudomonas* syringae type iii secreted effector proteins. *Front. Plant Sci.* 10:418. 10.3389/fpls.2019.00418 31024592 PMC6460904

[B19] Donaldson L. Meier S. Gehring C. (2016). The arabidopsis cyclic nucleotide interactome. *Cell Commun. Signal.* 14:10. 10.1186/s12964-016-0133-2 27170143 PMC4865018

[B20] Dyer M. D. Murali T. M. Sobral B. W. (2007). Computational prediction of host-pathogen protein-protein interactions. *Bioinformatics* 23 i159–i166. 10.1093/bioinformatics/btm208 17646292

[B21] Eddy S. R. (2011). Accelerated profile HMM searches. *PLoS Comput. Biol.* 7:e1002195. 10.1371/journal.pcbi.1002195 22039361 PMC3197634

[B22] Eichmann R. Schäfer P. (2012). The endoplasmic reticulum in plant immunity and cell death. *Front. Plant Sci.* 3:200. 10.3389/fpls.2012.00200 22936941 PMC3424470

[B23] Feil H. Feil W. S. Chain P. Larimer F. DiBartolo G. Copeland A. (2005). Comparison of the complete genome sequences of *Pseudomonas* syringae pv. syringae B728a and pv. tomato DC3000. *Proc. Natl. Acad. Sci. U.S.A.* 102 11064–11069. 10.1073/pnas.0504930102 16043691 PMC1182459

[B24] Finn R. D. Bateman A. Clements J. Coggill P. Eberhardt R. Y. Eddy S. R. (2014). Pfam: the protein families database. *Nucleic Acids Res.* 42 222–230. 10.1093/nar/gkt1223 24288371 PMC3965110

[B25] Fu L. Niu B. Zhu Z. Wu S. Li W. (2012). CD-HIT: accelerated for clustering the next-generation sequencing data. *Bioinformatics* 28 3150–3152. 10.1093/bioinformatics/bts565 23060610 PMC3516142

[B26] Grudniak A. M. Markowska K. Wolska K. I. (2015). Interactions of *Escherichia coli* molecular chaperone HtpG with DnaA replication initiator DNA. *Cell Stress Chaperones* 20 951–957. 10.1007/s12192-015-0623-y 26246199 PMC4595432

[B27] Guo Q. Q. Zhang W. B. Zhang C. Song Y. L. Liao Y. L. Ma J. C. (2019). Characterization of 3-oxacyl-acyl carrier protein reductase homolog genes in *Pseudomonas aeruginosa* PAO1. *Front. Microbiol.* 10:1028. 10.3389/fmicb.2019.01028 31231314 PMC6558427

[B28] Harighi B. (2007). Occurrence of alfalfa bacterial stem blight disease in Kurdistan province, Iran. *J. Phytopathol.* 155 593–595. 10.1111/j.1439-0434.2007.01284.x

[B29] Hu Y. (2006). Efficient, high-quality force-directed graph drawing. *Math. J.* 10 37–71.

[B30] Jagessar K. L. Jain C. (2010). Functional and molecular analysis of *Escherichia coli* strains lacking multiple DEAD-box helicases. *RNA* 16 1386–1392. 10.1261/rna.2015610 20484467 PMC2885687

[B31] Jasiński M. Stukkens Y. Degand H. Purnelle B. Marchand-Brynaert J. Boutry M. (2001). A plant plasma membrane ATP binding cassette-type transporter is involved in antifungal terpenoid secretion. *Plant Cell* 13 1095–1107. 10.1105/tpc.13.5.1095 11340184 PMC135550

[B32] Jehl M. A. Arnold R. Rattei T. (2011). Effective-A database of predicted secreted bacterial proteins. *Nucleic Acids Res.* 39 591–595. 10.1093/nar/gkq1154 21071416 PMC3013723

[B33] Jelenska J. Van Hal J. A. Greenberg J. T. (2010). *Pseudomonas* syringae hijacks plant stress chaperone machinery for virulence. *Proc. Natl. Acad. Sci. U.S.A.* 107 13177–13182. 10.1073/pnas.0910943107 20615948 PMC2919979

[B34] Kang J. Park J. Choi H. Burla B. Kretzschmar T. Lee Y. (2011). Plant ABC Transporters. *Arab. B.* 9:e0153. 10.1199/tab.0153 22303277 PMC3268509

[B35] Kerrien S. Aranda B. Breuza L. Bridge A. Broackes-Carter F. Chen C. (2012). The IntAct molecular interaction database in 2012. *Nucleic Acids Res.* 40 841–846. 10.1093/nar/gkr1088 22121220 PMC3245075

[B36] Keskin O. Nussinov R. Gursoy A. (2008). Prism: protein-protein interaction prediction by structural matching. *Methods Mol. Biol.* 484 505–521. 10.1007/978-1-59745-398-1_30 18592198 PMC2685641

[B37] Kim Y. Min B. Yi G. S. (2012). IDDI: integrated domain-domain interaction and protein interaction analysis system. *Proteome Sci.* 10:S9. 10.1186/1477-5956-10-S1-S9 22759586 PMC3380739

[B38] Kominek J. Marszalek J. Neuvéglise C. Craig E. A. Williams B. L. (2013). The complex evolutionary dynamics of Hsp70s: a genomic and functional perspective. *Genome Biol. Evol.* 5 2460–2477. 10.1093/gbe/evt192 24277689 PMC3879978

[B39] Kopittke P. M. (2016). Role of phytohormones in aluminium rhizotoxicity. *Plant Cell Environ.* 39 2319–2328. 10.1111/pce.12786 27352002

[B40] Kretschmer M. Damoo D. Djamei A. Kronstad J. (2020). Chloroplasts and plant immunity: where are the fungal effectors? *Pathogens* 9:19. 10.3390/pathogens9010019 31878153 PMC7168614

[B41] Kumar R. Nanduri B. (2010). HPIDB - a unified resource for host-pathogen interactions. *BMC Bioinformatics* 11:S16. 10.1186/1471-2105-11-16 20946599 PMC3026363

[B42] Kurubanjerdjit N. Tsai J. J. P. Sheu C. Y. Ng K. L. (2013). The prediction of protein-protein interaction of A. thaliana and X. campestris pv. campestris based on protein domain and interolog approaches. *Plant Omics* 6 388–398.

[B43] Kuzmanov U. Emili A. (2013). Protein-protein interaction networks: probing disease mechanisms using model systems. *Genome Med.* 5:37. 10.1186/gm441 23635424 PMC3706760

[B44] Lamichhane J. R. Messéan A. Morris C. E. (2015). Insights into epidemiology and control of diseases of annual plants caused by the *Pseudomonas* syringae species complex. *J. Gen. Plant Pathol.* 81 331–350. 10.1007/s10327-015-0605-z

[B45] Li D. Liu H. Zhang H. Wang X. Song F. (2008). OsBIRH1, a DEAD-box RNA helicase with functions in modulating defence responses against pathogen infection and oxidative stress. *J. Exp. Bot.* 59 2133–2146. 10.1093/jxb/ern072 18441339 PMC2413282

[B46] Li Q. Yan Q. Chen J. He Y. Wang J. Zhang H. (2012). Molecular characterization of an ice nucleation protein variant (InaQ) from *Pseudomonas* syringae and the analysis of its transmembrane transport activity in *Escherichia coli*. *Int. J. Biol. Sci.* 8 1097–1108. 10.7150/ijbs.4524 22991498 PMC3445048

[B47] Li Z. G. He F. Zhang Z. Peng Y. L. (2012). Prediction of protein-protein interactions between *Ralstonia solanacearum* and *Arabidopsis thaliana*. *Amino Acids* 42 2363–2371. 10.1007/s00726-011-0978-z 21786137

[B48] Lian X. Yang X. Shao J. Hou F. Yang S. Pan D. (2020). Prediction and analysis of human-herpes simplex virus type 1 protein-protein interactions by integrating multiple methods. *Quant. Biol.* 8 312–324. 10.21203/rs.2.22765/v1

[B49] Licata L. Briganti L. Peluso D. Perfetto L. Iannuccelli M. Galeota E. (2012). MINT, the molecular interaction database: 2012 update. *Nucleic Acids Res.* 40 857–861. 10.1093/nar/gkr930 22096227 PMC3244991

[B50] Lindow S. E. Lahue E. Govindarajan A. G. Panopoulos N. J. Gies D. (1989). Localization of ice nucleation activity and the iceC gene product in *Pseudomonas* syringae and *Escherichia coli*. *Mol. Plant. Microbe Interact.* 2 262–272. 10.1094/mpmi-2-262 2520825

[B51] Link T. Lohaus G. Heiser I. Mendgen K. Hahn M. Voegele R. T. (2005). Characterization of a novel NADP+-dependent D-arabitol dehydrogenase from the plant pathogen *Uromyces fabae*. *Biochem. J.* 389 289–295. 10.1042/BJ20050301 15796718 PMC1175105

[B52] Lipps S. M. Lenz P. Samac D. A. (2019). First report of bacterial stem blight of alfalfa caused by *Pseudomonas* viridiflava in California and Utah. *Plant Dis.* 103:3274. 10.1094/PDIS-05-19-1044-PDN

[B53] Liu J. X. Howell S. H. (2010). Endoplasmic reticulum protein quality control and its relationship to environmental stress responses in plants. *Plant Cell* 22 2930–2942. 10.1105/tpc.110.078154 20876830 PMC2965551

[B54] Loaiza C. D. Duhan N. Lister M. Kaundal R. (2020). In silico prediction of host–pathogen protein interactions in melioidosis pathogen Burkholderia pseudomallei and human reveals novel virulence factors and their targets. *Brief. Bioinform.* 22:bbz162. 10.1093/bib/bbz162 32444871

[B55] Lu Y. Yao J. (2018). Chloroplasts at the crossroad of photosynthesis, pathogen infection and plant defense. *Int. J. Mol. Sci.* 19:3900. 10.3390/ijms19123900 30563149 PMC6321325

[B56] Martin S. Brown W. M. Klavans R. Boyack K. W. (2011). “OpenOrd: an open-source toolbox for large graph layout,” in *Visualization and Data Analysis*, eds Wong P. C. Park J. Hao M. C. Chen C. Börner K. Kao D. L. (Bellingham, WA: SPIE), 45–55. 10.1117/12.871402

[B57] Matthews L. R. Vaglio P. Reboul J. Ge H. Davis B. P. Garrels J. (2001). Identification of potential interaction networks using sequence-based searches for conserved protein-protein interactions or “interologs.”. *Genome Res.* 11 2120–2126. 10.1101/gr.205301 11731503 PMC311221

[B58] Millyard L. Lee J. Zhang C. Yates G. Sadanandom A. (2016). The ubiquitin conjugating enzyme, TaU4 regulates wheat defence against the phytopathogen Zymoseptoria tritici. *Sci. Rep.* 6:35683. 10.1038/srep35683 27759089 PMC5069635

[B59] Mondal S. I. Mahmud Z. Elahi M. Akter A. Jewel N. A. Muzahidul Islam M. (2017). Study of intra–inter species protein–protein interactions for potential drug targets identification and subsequent drug design for *Escherichia coli* O104:H4 C277-11. *Silico Pharmacol.* 5:1. 10.1007/s40203-017-0021-5 28401513 PMC5391048

[B60] Morris C. E. Monteil C. L. Berge O. (2013). The life history of *pseudomonas* syringae: linking agriculture to earth system processes. *Annu. Rev. Phytopathol.* 51 85–104. 10.1146/annurev-phyto-082712-102402 23663005

[B61] Mosca R. Céol A. Stein A. Olivella R. Aloy P. (2014). 3did: a catalog of domain-based interactions of known three-dimensional structure. *Nucleic Acids Res.* 42 374–379. 10.1093/nar/gkt887 24081580 PMC3965002

[B62] Nemchinov L. G. Shao J. Lee M. N. Postnikova O. A. Samac D. A. (2017). Resistant and susceptible responses in alfalfa (*Medicago sativa*) to bacterial stem blight caused by *Pseudomonas* syringae pv. syringae. *PLoS One* 12:e0189781. 10.1371/journal.pone.0189781 29244864 PMC5731681

[B63] Ng S. K. Zhang Z. Tan S. H. (2003). Integrative approach for computationally inferring protein domain interactions. *Bioinformatics* 19 923–929. 10.1093/bioinformatics/btg118 12761053

[B64] Nühse T. S. Peck S. C. Hirt H. Boller T. (2000). Microbial elicitors induce activation and dual phosphorylation of the Arabidopsis thaliana MAPK 6. *J. Biol. Chem.* 275 7521–7526. 10.1074/jbc.275.11.7521 10713056

[B65] Olsen J. V. Blagoev B. Gnad F. Macek B. Kumar C. Mortensen P. (2006). Global, in vivo, and site-specific phosphorylation dynamics in signaling networks. *Cell* 127 635–648. 10.1016/j.cell.2006.09.026 17081983

[B66] Park C. J. Caddell D. F. Ronald P. C. (2012). Protein phosphorylation in plant immunity: insights into the regulation of pattern recognition receptor-mediated signaling. *Front. Plant Sci.* 3:177. 10.3389/fpls.2012.00177 22876255 PMC3411088

[B67] Park E. Nedo A. Caplan J. L. Dinesh-Kumar S. P. (2018). Plant–microbe interactions: organelles and the cytoskeleton in action. *New Phytol.* 217 1012–1028. 10.1111/nph.14959 29250789

[B68] Parthasarathy A. Savka M. A. Hudson A. O. (2019). The synthesis and role of β-alanine in plants. *Front. Plant Sci.* 10:921. 10.3389/fpls.2019.00921 31379903 PMC6657504

[B69] Pavlopoulos G. A. Paez-Espino D. Kyrpides N. C. Iliopoulos I. (2017). Empirical comparison of visualization tools for larger-scale network analysis. *Adv. Bioinformatics* 2017:1278932. 10.1155/2017/1278932 28804499 PMC5540468

[B70] Pelgrom A. J. E. Meisrimler C. N. Elberse J. Koorman T. Boxem M. van den Ackerveken G. (2020). Host interactors of effector proteins of the lettuce downy mildew *Bremia lactucae* obtained by yeast two-hybrid screening. *PLoS One* 15:e0226540. 10.1371/journal.pone.0226540 32396563 PMC7217486

[B71] Proud sponsor of Midwest Forage Association (2017). *Proud sponsor of Midwest Forage Association.* Available online at: https://www.ars.usda.gov/ARSUserFiles/4909/Updates/2017_Samac_BacterialStem.pdf (accessed October 19, 2020).

[B72] Pusztahelyi T. Holb I. J. Pócsi I. (2015). Secondary metabolites in fungus-plant interactions. *Front. Plant Sci.* 6:573. 10.3389/fpls.2015.00573 26300892 PMC4527079

[B73] Putnam D. H. Orloff S. B. (2014). “Forage crops,” in *Encyclopedia of Agriculture and Food Systems*, ed. Van Alfen N. K. (Oxford: Academic Press), 381–405.

[B74] Race A. Bultreys A. Kaluzna M. (2006). Supplement 1: recent advances in the study of bacterial diseases of stone fruit and nut trees: minireviews based on the activity of ESF. *J. Plant Pathol.* 92 S1.57–S1.51.

[B75] Raghavachari B. Tasneem A. Przytycka T. M. Jothi R. (2008). DOMINE: a database of protein domain interactions. *Nucleic Acids Res.* 36 656–661. 10.1093/nar/gkm761 17913741 PMC2238965

[B76] Rojas C. M. Senthil-Kumar M. Tzin V. Mysore K. S. (2014). Regulation of primary plant metabolism during plant-pathogen interactions and its contribution to plant defense. *Front. Plant Sci.* 5:17. 10.3389/fpls.2014.00017 24575102 PMC3919437

[B77] Sahu S. S. Loaiza C. D. Kaundal R. (2020). Plant-mSubP: a computational framework for the prediction of single- and multi-target protein subcellular localization using integrated machine-learning approaches. *AoB Plants* 12 1–10. 10.1093/aobpla/plz068 32528639 PMC7274489

[B78] Sahu S. S. Weirick T. Kaundal R. (2014). Predicting genome-scale Arabidopsis-*Pseudomonas* syringae interactome using domain and interolog-based approaches. *BMC Bioinformatics* 15:S13. 10.1186/1471-2105-15-S11-S13 25350354 PMC4251041

[B79] Salwinski L. Miller C. S. Smith A. J. Pettit F. K. Bowie J. U. Eisenberg D. (2004). The database of interacting proteins: 2004 update. *Nucleic Acids Res.* 32 449–451. 10.1093/nar/gkh086 14681454 PMC308820

[B80] Scheideler M. Schlaich N. L. Fellenberg K. Beissbarth T. Hauser N. C. Vingron M. (2002). Monitoring the switch from housekeeping to pathogen defense metabolism in *Arabidopsis thaliana* using cDNA arrays. *J. Biol. Chem.* 277 10555–10561. 10.1074/jbc.M104863200 11748215

[B81] Schepetilnikov M. Ryabova L. A. (2017). Auxin signaling in regulation of plant translation reinitiation. *Front. Plant Sci.* 8:1014. 10.3389/fpls.2017.01014 28659957 PMC5469914

[B82] Seo S. Okamoto M. Seto H. Ishizuka K. Sano H. Ohashi Y. (1995). Tobacco MAP kinase: a possible mediator in wound signal transduction pathways. *Science* 270 1988–1992. 10.1126/science.270.5244.1988 8533090

[B83] Shoemaker B. A. Panchenko A. R. (2007). Deciphering protein-protein interactions. Part II. Computational methods to predict protein and domain interaction partners. *PLoS Comput. Biol.* 3:e43. 10.1371/journal.pcbi.0030043 17465672 PMC1857810

[B84] Srivastava R. Chen Y. Deng Y. Brandizzi F. Howell S. H. (2012). Elements proximal to and within the transmembrane domain mediate the organelle-to-organelle movement of bZIP28 under ER stress conditions. *Plant J.* 70 1033–1042. 10.1111/j.1365-313X.2012.04943.x 22335396

[B85] Stahl E. A. Bishop J. G. (2000). Plant-pathogen arms races at the molecular level. *Curr. Opin. Plant Biol.* 3 299–304. 10.1016/S1369-5266(00)00083-2 10873849

[B86] Steimer L. Klostermeier D. (2012). RNA helicases in infection and disease. *RNA Biol.* 9 751–771. 10.4161/rna.20090 22699555

[B87] Sun T. Zhou B. Lai L. Pei J. (2017). Sequence-based prediction of protein protein interaction using a deep-learning algorithm. *BMC Bioinformatics* 18:277. 10.1186/s12859-017-1700-2 28545462 PMC5445391

[B88] Szklarczyk D. Gable A. L. Lyon D. Junge A. Wyder S. Huerta-Cepas J. (2019). STRING v11: protein-protein association networks with increased coverage, supporting functional discovery in genome-wide experimental datasets. *Nucleic Acids Res.* 47 D607–D613. 10.1093/nar/gky1131 30476243 PMC6323986

[B89] Thanasomboon R. Kalapanulak S. Netrphan S. Saithong T. (2017). Prediction of cassava protein interactome based on interolog method. *Sci. Rep.* 7:17206. 10.1038/s41598-017-17633-2 29222529 PMC5722940

[B90] Ul Haq S. Khan A. Ali M. Khattak A. M. Gai W. X. Zhang H. X. (2019). Heat shock proteins: dynamic biomolecules to counter plant biotic and abiotic stresses. *Int. J. Mol. Sci.* 20:5321. 10.3390/ijms20215321 31731530 PMC6862505

[B91] Urade R. (2009). The endoplasmic reticulum stress signaling pathways in plants. *Biofactors* 35 326–331. 10.1002/biof.45 19415737

[B92] Vela-Corcía D. Aditya Srivastava D. Dafa-Berger A. Rotem N. Barda O. Levy M. (2019). MFS transporter from Botrytis cinerea provides tolerance to glucosinolate-breakdown products and is required for pathogenicity. *Nat. Commun.* 10:2886. 10.1038/s41467-019-10860-3 31253809 PMC6599007

[B93] Wu X. Zhu L. Guo J. Zhang D. Y. Lin K. (2006). Prediction of yeast protein-protein interaction network: insights from the gene ontology and annotations. *Nucleic Acids Res.* 34 2137–2150. 10.1093/nar/gkl219 16641319 PMC1449908

[B94] Xu Z. Escamilla-Treviño L. L. Zeng L. Lalgondar M. Bevan D. R. Winkel B. S. J. (2004). Functional genomic analysis of *Arabidopsis thaliana* glycoside hydrolase family 1. *Plant Mol. Biol.* 55 343–367. 10.1007/s11103-004-0790-1 15604686

[B95] Xue L. Tang B. Chen W. Luo J. (2019). DeepT3: deep convolutional neural networks accurately identify gram-negative bacterial type III secreted effectors using the N-Terminal sequence. *Bioinformatics* 35 2051–2057. 10.1093/bioinformatics/bty931 30407530

[B96] Yamada K. Hara-Nishimura I. Nishimura M. (2011). Unique defense strategy by the endoplasmic reticulum body in plants. *Plant Cell Physiol.* 52 2039–2049. 10.1093/pcp/pcr156 22102697

[B97] Yang S. Li H. He H. Zhou Y. Zhang Z. (2019). Critical assessment and performance improvement of plant-pathogen protein-protein interaction prediction methods. *Brief. Bioinform.* 20 274–287. 10.1093/bib/bbx123 29028906

[B98] Yu A. Li P. Tang T. Wang J. Chen Y. Liu L. (2015). Roles of Hsp70s in stress responses of microorganisms, plants, and animals. *Biomed. Res. Int.* 2015:510319. 10.1155/2015/510319 26649306 PMC4663327

[B99] Yu G. Wang L. G. Han Y. He Q. Y. (2012). ClusterProfiler: an R package for comparing biological themes among gene clusters. *OMICS* 16 284–287. 10.1089/omi.2011.0118 22455463 PMC3339379

[B100] Yu H. Luscombe N. M. Lu H. X. Zhu X. Xia Y. Han J. D. J. (2004). Annotation transfer between genomes: protein-protein interrologs and protein-DNA regulogs. *Genome Res.* 14 1107–1118. 10.1101/gr.1774904 15173116 PMC419789

[B101] Yu N. Y. Wagner J. R. Laird M. R. Melli G. Rey S. Lo R. (2010). PSORTb 3.0: improved protein subcellular localization prediction with refined localization subcategories and predictive capabilities for all prokaryotes. *Bioinformatics* 26 1608–1615. 10.1093/bioinformatics/btq249 20472543 PMC2887053

[B102] Zalguizuri A. Caetano-Anollés G. Lepek V. C. (2018). Phylogenetic profiling, an untapped resource for the prediction of secreted proteins and its complementation with sequence-based classifiers in bacterial type III, IV and VI secretion systems. *Brief. Bioinform.* 20 1395–1402. 10.1093/bib/bby009 29394318

[B103] Zeng Y. Charkowski A. O. (2020). The role of ATP-binding cassette (ABC) transporters in bacterial phytopathogenesis. *Phytopathology* 111 600–610. 10.1094/phyto-06-20-0212-rvw 33225831

[B104] Zhang S. Klessig D. F. (1997). Salicylic acid activates a 48-kD MAP kinase in tobacco. *Plant Cell* 9 809–824. 10.1105/tpc.9.5.809 9165755 PMC156958

[B105] Zhong X. Rajapakse J. C. (2020). Graph embeddings on gene ontology annotations for protein–protein interaction prediction. *BMC Bioinformatics* 21:560. 10.1186/s12859-020-03816-8 33323115 PMC7739483

[B106] Ziehe D. Dünschede B. Schünemann D. (2017). From bacteria to chloroplasts: evolution of the chloroplast SRP system. *Biol. Chem.* 398 653–661. 10.1515/hsz-2016-0292 28076289

[B107] Zurbriggen M. D. Carrillo N. Tognetti V. B. Melzer M. Peisker M. Hause B. (2009). Chloroplast-generated reactive oxygen species play a major role in localized cell death during the non-host interaction between tobacco and *Xanthomonas campestris* pv. vesicatoria. *Plant J.* 60 962–973. 10.1111/j.1365-313X.2009.04010.x 19719480

