# Supplementary material for: Computational Systems Biology of Alfalfa – Bacterial Blight Host-Pathogen Interactions: Uncovering the Complex Molecular Networks for Developing Durable Disease Resistant Crop
Source: Front Plant Sci. 2022 Feb 17;12:807354. doi: 10.3389/fpls.2021.807354 (PMC8891223; doi:10.3389/fpls.2021.807354)
Supplement: Supplementary Figure 1 — Top 15 P. syringae GO terms that were found over-represented based on enrichment score [−log 10(P-value)]: Molecular function (blue), cellular component (green), and biological process (red). [file Table_1.DOCX]

**Supplementary Material**

***Computational systems biology of Alfalfa - Bacterial Blight host-pathogen interactions: uncovering the complex molecular networks for developing durable disease resistant crop***

There are 7 Excel files that contains the supplementary material. In addition, there are 7 supplementary figures. Due to the overall file size limitations on the Frontiers website, all these 7 excel files (and 7 suppl figures) are available for access at <http://biocluster.usu.edu/publications/rkataria/alfalfa_PPIs/Supplementary_Material>

Thank you.
